# Supplementary material for: The interactome of intact mitochondria by cross-linking mass spectrometry provides evidence for coexisting respiratory supercomplexes
Source: Mol Cell Proteomics. 2017 Dec 8;17(2):216–32. doi: 10.1074/mcp.RA117.000470 (PMC5795388; doi:10.1074/mcp.RA117.000470)
Supplement: Supplemental Data [file supp_RA117.000470_133922_0_supp_23978_fzffwf.zip › spectra_annotation/mito_spectra_annotation/167-1-12-1-8-1.pdf]

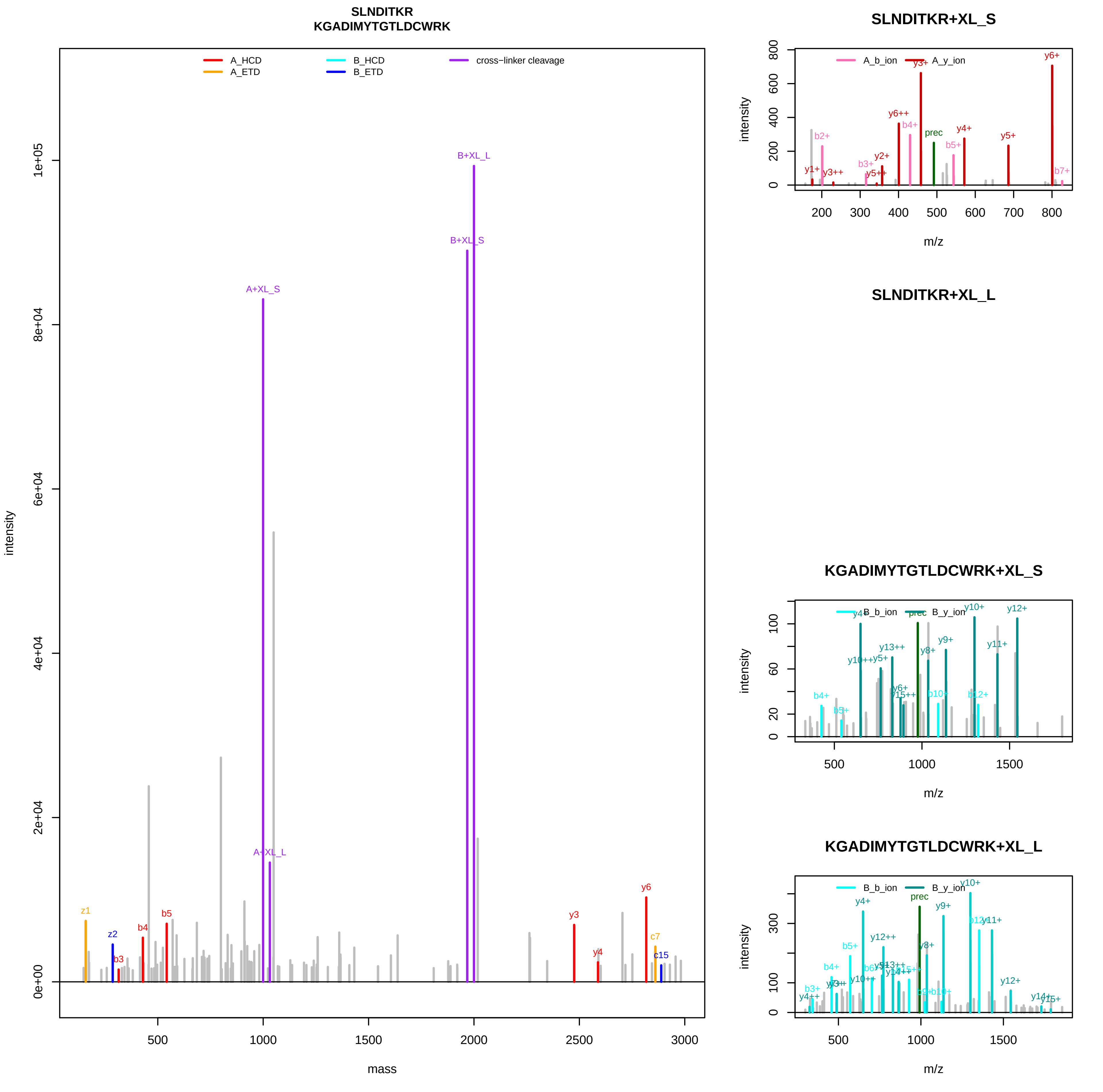
SLNDITKR
KGADIMYTGTLDCWRK
A_HCD
A_ETD
B_HCD
B_ETD
cross−linker cleavage
intensity
mass
0e+00
2e+04
4e+04
6e+04
8e+04
1e+05
500
1000
1500
2000
2500
3000
B+XL_L
B+XL_S
A+XL_S
A+XL_L
z1
z2
b3
b4
b5
y3
y4
y6
c7
c15
SLNDITKR+XL_S
A_b_ion
A_y_ion
intensity
m/z
y1+
b2+
y3++
b3+
y5++
y2+
y6++
b4+
y3+
prec
b5+
y4+
y5+
y6+
b7+
SLNDITKR+XL_L
KGADIMYTGTLDCWRK+XL_S
B_b_ion
B_y_ion
intensity
m/z
y4+
prec
y10+
y12+
y13++
y10++
y5+
y8+
y9+
y11+
b4+
b5+
y6+
b10+
b12+
KGADIMYTGTLDCWRK+XL_L
B_b_ion
B_y_ion
intensity
m/z
y10+
y4+
prec
y9+
y12++
b5+
y8+
b12+
y11+
b4+
y13++
y14++
y10++
b3+
y4++
y3+
y12+
y14+
y15+
